# Neuropsychiatric Symptoms in Comorbid Alzheimer's Disease Neuropathologic Change and Frontotemporal Lobar Degeneration

**DOI:** 10.1002/alz70857_097441

**Published:** 2025-12-24

**Authors:** Daliah Ross, Molly Split, Zachary J. Kunicki, Rachel M Keszycki, Sarah Prieto, Masood Manoochehri, Edward D. Huey, Megan S Barker

**Affiliations:** ^1^ Brown University, Providence, RI, USA; ^2^ Butler Hospital, Providence, RI, USA; ^3^ Northwestern University Feinberg School of Medicine, Chicago, IL, USA; ^4^ Mesulam Center for Cognitive Neurology & Alzheimer's Disease, Chicago, IL, USA; ^5^ Department of Psychiatry and Human Behavior, Alpert Medical School of Brown University, Providence, RI, USA

## Abstract

**Background:**

Neurodegenerative diseases often co‐occur; however, little is known about the clinical presentation of comorbid Alzheimer's disease neuropathologic change (ADNC) and frontotemporal lobar degeneration (FTLD) neuropathology despite frequent comorbidity on autopsy. We examined neuropsychiatric symptoms in comorbid FTLD (tau or TDP‐43) and AD, compared to FTLD and AD pathology alone.

**Method:**

National Alzheimer's Coordinating Center data from 29 Alzheimer's Disease Research Centers (as of September 2024) was used to compare antemortem neuropsychiatric data in 919 people with intermediate to high ADNC, FTLD‐tau and/or FTLD‐TDP‐43 neuropathology and clinical data at their last visit. Neuropsychiatric symptoms were identified via clinician‐rated presence or absence of apathy/withdrawal, depressed mood, visual/auditory hallucinations, abnormal/false/delusional beliefs, disinhibition, irritability, agitation, personality change, REM sleep behavior disorder, and anxiety at the final visit before death. We ran logistic regression models to examine odds of expressing each neuropsychiatric symptom by pathology, controlling for age, sex, race, ethnicity, education, and time between visit and death.

**Result:**

94 people (mean age=84, *SD* = 10, 46% female) had comorbid ADNC and FTLD pathology, 590 people had ADNC only, and 235 people had FTLD pathology (65% tau, 29% TDP‐43, 7% both types) only. Compared to the comorbid ADNC and FTLD group, the FTLD‐only group was less likely to present with delusions (O.R. = 0.38, *p* = .021), irritability (O.R. = 0.54, *p* = .028), and anxiety (O.R. = 0.33, *p* = .004). Conversely, the ADNC‐only group was less likely to present with disinhibition (O.R. = 0.50, *p* = .017) and personality change (O.R. = 0.32, *p* < .001). Likelihood of presenting with apathy, depression, hallucinations, agitation, and REM sleep behavior did not differ in the comorbid group compared to either single pathology group (*p*s > .05).

**Conclusion:**

Comorbid presence of ADNC and FTLD neuropathology was associated with greater likelihood of presenting with known neuropsychiatric symptoms of the other disease, irrespective of clinical syndrome. Disinhibition and personality change differentiated comorbid ADNC and FTLD from ADNC alone, while delusions, irritability, and anxiety differentiated comorbid ADNC and FTLD from FTLD alone. These findings highlight the diagnostic value of antemortem behavioral symptoms in comorbid pathology. Moreover, findings are consistent with well‐established neuropsychiatric phenotypes of ADNC and FTLD, emphasizing the importance of clinical phenotypes in precision medicine.

